# The moderating effects of physical activity on social anxiety and sleep disturbance: managing gaming disorder in young e-sports players

**DOI:** 10.3389/fpubh.2025.1544044

**Published:** 2025-02-06

**Authors:** Serdar Solmaz, Mehmet İnan, Mustafa Yaşar Şahin

**Affiliations:** ^1^Department of Sport Management, Faculty of Sport Sciences, Batman University, Batman, Türkiye; ^2^Department of Coaching Education, Faculty of Sport Sciences, Bozok University, Yozgat, Türkiye; ^3^Department of Sport Management, Faculty of Sport Sciences, Gazi University, Ankara, Türkiye

**Keywords:** e-sports, gaming addiction, mental health, active lifestyle, youth and adolescents

## Abstract

**Background:**

Internet Gaming Disorder (IGD) has emerged as an increasing public health concern, particularly among adolescent and young e-sports players in recent years. The immersive and competitive nature of online gaming has been associated with an increase in mental health issues such as anxiety and insomnia among individuals with IGD. This study aims to examine the potential relationship between IGD, social anxiety, and insomnia, investigate the mediating role of social anxiety in this relationship, and explore the moderating role of physical activity in alleviating the negative effects of IGD.

**Methods:**

The study involved 475 adolescent and young e-sports players aged 14–24, who typically engage in an average of 6 h of gaming per day. Participants completed the Internet Gaming Disorder Scale-Short Form, Social Anxiety Inventory, Insomnia Severity Index, and Physical Activity Frequency Question. Correlation and moderation analyses were used to examine the proposed relationships.

**Results:**

The findings revealed significant positive associations between IGD, social anxiety, and insomnia. Moreover, physical activity demonstrated a negative correlation with both social anxiety and insomnia. Notably, physical activity served as a moderator in the relationship between IGD and both social anxiety and insomnia, with higher levels of physical activity attenuating the adverse effects of IGD.

**Conclusion:**

This study illuminates the intricate relationship between IGD, social anxiety, and insomnia among adolescent and young e-sports players. The findings suggest that promoting physical activity may alleviate the negative psychological implications associated with IGD. These results provide important insights for the formulation of targeted intervention strategies aimed at this demographic.

## 1 Introduction

E-sports has rapidly emerged as a competitive digital domain, attracting significant interest from adolescents and young individuals in recent years (1, 2). Representing a generation born into the digital age, young people view e-sports not only as a source of entertainment but also as a platform for shaping their social identity and career aspirations (3, 4). While engagement in digital gaming can enhance motor skills (5) and overall wellbeing in the short term (6), it may lead to severe physical and psychological health issues in the long term (7–9). In this context, IGD, as defined by the American Psychiatric Association (APA) in the DSM-5-TR, is described as a disorder caused by persistent and excessive internet use, which can result in clinically significant health problems and requires further research (10). Similarly, the WHO recognized Gaming Disorder as an official health condition in 2019, emphasizing its potential adverse effects as a global public health issue (11). Particularly during adolescence and young adulthood, a critical period for identity formation and behavioral development (12), —IGD has been associated with negative impacts on daily life quality and cognitive functioning, posing serious mental and physical health risks that threaten public wellbeing (2, 7).

This study examines the increasing issues of insomnia (13) and social anxiety (14) observed among young and adolescent e-sports players as a consequence of IGD, with a focus on the potential moderating role of regular physical activity. While insomnia is a common problem associated with digital game addiction (15, 16), social anxiety is a critical factor that adversely affects the mental health of young individuals (17). The limited number of studies addressing these complex relationships collectively enhances the originality and significance of this research. In this context, focusing on the protective role of physical activity among young e-sports players (18), addresses a critical gap in the literature by contributing to the development of strategies that help young individuals cope with digital addiction. Promoting the establishment of strong social relationships and fostering both physically and mentally healthy individuals—who will shape the future of societies—can only be achieved through interventions initiated during this developmental stage. The aim of this study is to investigate the effects of IGD, which is becoming increasingly prevalent among young individuals, on negative outcomes such as social anxiety and insomnia, while exploring how physical activity moderates these relationships.

### 1.1 The mediating role of social anxiety

IGD is a condition that can lead to disruptions in individuals’ social and psychological functioning due to difficulties in controlling their gaming habits (10, 11). According to Social Cognitive Theory (19) and Clark and Wells (17) model of social anxiety, low social cognitive skills and low self-esteem can lead to an increase in social anxiety levels. IGD can trigger these conditions, paving the way for elevated social anxiety (14). Young and adolescent individuals who spend extended periods in online environments experience limited social interactions and begin to struggle with face-to-face relationships. With withdrawal from their social circles, these individuals’ social skills fail to develop, and the deficiencies in these skills become more pronounced over time. This becomes a factor that increases social anxiety. Additionally, since online games are often based on individual or virtual interactions, young people are less exposed to real-world social norms and empathy skills. These deficiencies lead them to feel insecure in social settings and fear that others will negatively evaluate them. As a result, these individuals may tend to avoid social interactions, which reinforces social anxiety. Furthermore, cognitive issues caused by IGD, such as attention deficits and stress, impair the individual’s ability to regulate themselves during social interactions, thus increasing social anxiety. All these factors together initiate the process by which IGD triggers social anxiety (2, 20).

Individuals with social anxiety experience intense feelings of anxiety due to the fear of being negatively evaluated by others, and they tend to cope with these situations through avoidance (17). This avoidance behavior leads to social withdrawal and continuous preoccupation with their anxiety. The persistent focus on anxious thoughts can elevate stress levels, which, over time, may contribute to more complex mental health issues, such as depression (21, 22).These mental health problems, particularly stress and depression, can have negative effects on both biological and psychological systems, leading to disruptions in sleep patterns (14, 22). Considering that social anxiety may cause individuals to avoid social interactions, and that this isolation can result in an accumulation of heightened anxiety and stress over time (2, 20), it can be concluded that there is a direct relationship between social anxiety and sleep disturbances. Specifically, young e-sports players affected by such anxiety may experience sleep problems, such as waking up during the night or experiencing poor sleep quality.

Based on these findings and theoretical frameworks, it can be hypothesized that IGD may influence social anxiety in young and adolescent individuals who play e-sports, and that social anxiety, in turn, may contribute to the development of insomnia symptoms. Therefore, the present study hypothesizes that social anxiety will mediate the relationship between IGD and sleep disorders (H1).

### 1.2 The moderating effect of physical activity

Physical activity is not only crucial for supporting physical health, but also plays a significant role in improving mental and emotional wellbeing, contributing to an overall increase in an individual’s quality of life (23). Particularly during adolescence and young adulthood, regular physical activity enhances individuals’ ability to cope with stress, supports emotional regulation, and fosters social interactions (23, 24). These effects make physical activity even more important during this critical developmental period (25), which is characterized by rapid physical, social, and psychological changes.

Physical activities naturally stimulate the production of endorphins in the brain, which can promote social bonding, alleviate pain, and improve mood. As a result, feelings of anxiety and unhappiness may decrease (23, 24). Indeed, systematic review studies on adolescents and young individuals have observed that physical activity and various exercises alleviate symptoms of depression and anxiety (23, 26). According to Social Cognitive Theory, self-efficacy is not only a concept related to coping with stress, but also directly influences psychosomatic regulatory systems, shaping mental health (27). According to self-efficacy theory, regular physical activity can enhance the self-efficacy of young individuals (27, 28), and those with higher self-efficacy can reduce the fear of negative evaluation, thereby alleviating social anxiety symptoms (29). A systematic review and meta-analysis have reported significant effects of physical activity on anxiety and social anxiety (30).

Studies conducted at different times and in various geographical locations have concluded that young individuals with internet addiction and those who play e-sports experience poor sleep quality and insomnia (15, 16). Physical activity and exercise are recommended as alternative, non-pharmacological treatment options for sleep regulation (31). Results from various studies have shown that physically active adolescents have better sleep quality compared to those who are inactive (32, 33). In general, physical activity in young and adolescent individuals can be considered a non-pharmacological treatment method that significantly reduces the severity of insomnia symptoms.

**Figure 1:**
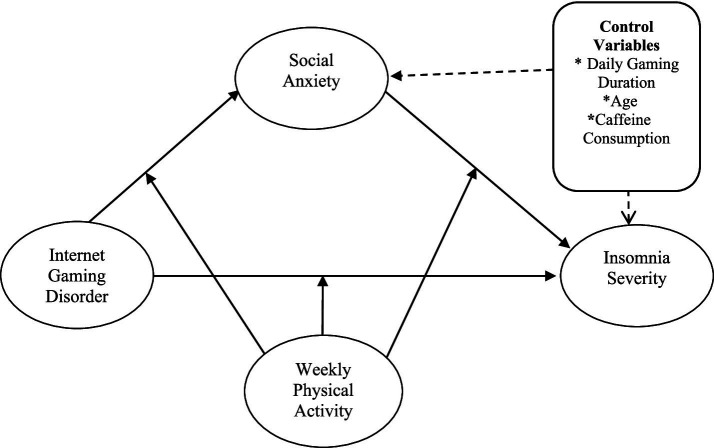
Hypothesized conceptual model.

Based on theoretical frameworks and previous studies, it is hypothesized that physical activity will moderate the effects of IGD on social anxiety and insomnia, as well as the effect of social anxiety on insomnia (H2). Compared to e-sports players with low participation in physical activity, those with high participation in physical activity are expected to have lower levels of social anxiety and insomnia (Figure 1). Additionally, in the proposed model, control variables including daily gaming duration, age, and caffeine consumption, which have been shown to influence social anxiety and insomnia, have been added to the model (34–36). After the inclusion of control variables, it is expected that the relationship between IGD and insomnia will remain significant in the current study.

## 2 Methodology

### 2.1 Participants and procedures

In the study, collaborations were established with e-sports influencers on popular digital platforms targeting a young and broad audience to reach the necessary sample size. To encourage young e-sports players to complete the survey prepared on Google Forms, raffle prizes were offered, with winners receiving vouchers—set at prices determined by the influencers—that could be used in the games they played. Participants who completed the survey had a chance to enter the raffle. This approach allowed us to reach a wider audience on digital platforms and obtain data from a more diverse participant group. Data were collected over 10 days from approximately 475 individuals; however, 72 participants were excluded from the analysis due to failing to meet the study criteria or providing inaccurate information. Specifically, participants were required to have been playing e-sports for at least 3 h per day and for a minimum of 6 months, in order to exclude seasonal or temporary factors. Additionally, individuals who reported having any diagnosed psychological disorders were excluded to reduce confounding factors and ensure the validity of the data. The administered surveys included the Internet Gaming Disorder Inventory, Social Anxiety Inventory, Insomnia Severity Index, and questions measuring Weekly Physical Activity levels.

The study sample consists of amateur young players aged between 14 and 24 who play e-sports games such as League of Legends, PUBG, Valorant, Fortnite, and Counter-Strike: Global Offensive. An examination of participants’ educational backgrounds revealed that 6.9% were middle school students, 45.7% were high school students, 43.2% were undergraduate students, and 4.2% were graduate students. Of the participants, 71.5% were male and 28.5% were female. Additionally, participants’ daily gaming duration ranged from 3 to 10 h.

The calculation was conducted based on the reference data reported by Lin et al. (37) to ensure an adequate sample size. In these calculations, the number of independent variables (*u* = 3), the anticipated effect size (*f*^2^ = 0.067), 95% statistical power, and an alpha level of 0.05 were considered; accordingly, the required sample size was determined to be 242 participants.

### 2.2 Measures

#### 2.2.1 Internet gaming disorder scale-short form (IGDS9-SF)

The Internet Gaming Disorder (IGD) symptom severity was measured using the IGDS9-SF, a 9-item short self-assessment scale developed by Pontes and Griffiths (38), based on DSM-5 criteria. The scale contains questions regarding gaming activities over the last year. Responses were collected using a 5-point Likert scale. Higher total scores indicate greater severity of IGD. The validity and reliability study of the scale in Turkish was conducted by Arıcak et al. (39), and demonstrated sufficient psychometric properties. For this study, McDonald’s Omega (*ω*) was calculated as 0.88.

#### 2.2.2 Social anxiety inventory (LSAS)

The Liebowitz Social Anxiety Scale was used to measure the severity of social anxiety symptoms (40). This scale assesses participants’ levels of fear and avoidance in specific social situations using a 4-point Likert-type rating scale ranging from 0 (none) to 3 (severe). The total score indicates the severity of social anxiety. The validity and reliability study of the scale in Turkish conducted by Soykan et al. (41) and demonstrated sufficient psychometric properties. In this study, McDonald’s Omega (*ω*) was calculated to be 0.95.

#### 2.2.3 Insomnia severity inventory (ISI)

The Insomnia Severity Index (ISI) scale was utilized to measure the severity of insomnia (42). Participants’ insomnia severity was assessed using a 5-point Likert scale ranging from 0 (none) to 4 (very severe). Higher scores indicate more severe insomnia symptoms. The validity and reliability study of the scale in Turkish was carried out by Boysan et al. (43) and demonstrated sufficient psychometric properties. This study calculated McDonald’s Omega (*ω*) to be 0.88.

#### 2.2.4 Physical activity (PA)

In the study, participants asked a single question about physical activity. They were presented with the question, “In the past week, on how many days have you done a total of 30 min or more of physical activity, which was enough to raise your breathing rate?” and asked to respond with the number of days as an open-ended answer. Although physical activity (PA) was assessed with a single question, it can provide acceptable reliability and construct validity in situations where it is not the primary focus of the study and more detailed measurements are not implemented (44, 45). Additionally, single-question measures of physical activity have been used by different researchers at various times and have demonstrated reliable results (45, 46).

### 2.3 Analytic strategy

In this study, which was conducted with a sample group of young e-sports players, Internet gaming disorder was modeled as the independent variable, insomnia severity as the dependent variable, and social anxiety as the mediating variable. Additionally, the weekly physical activity variable was included in the model as a moderator in the relationships among these variables. Path analysis was employed to determine the mediating and moderating effects (47). The Maximum Likelihood (ML) estimation method was used to calculate these effects. Standardized factor values and their z-scores were utilized to determine the significance levels of the variables in the study. In the study, the values recommended by Kline (48) for SEM analyses were reported, including *χ*^2^/df, the *p*-value associated with, 2 Comparative Fit Index (CFI), Root Mean Square Error of Approximation (RMSEA), and Standardized Root Mean Square Residual (SRMR). In this context, cut-off points were set as *χ*^2^/df < 3, CFI < 0.90, RMSEA <0.08, and SRMR <0.08 (49, 50). The bootstrap technique was employed to validate whether the established model and the relationships among the variables were statistically significant (51). In the current study, 5,000 resampling options were chosen and calculated within a 95% confidence interval (52).

The data analysis conducted in this study was performed using R Studio (4.0.5) and SPSS 26 software. Structural equation modeling and confirmatory factor analyses were carried out using the “lavaan” package, the “semTools” package, was used for confidence intervals, and the “pwr” package was employed for sample size calculations.

## 3 Findings

### 3.1 Evaluation of the measurement model

Descriptive statistics, correlations, and reliabilities for the variables of the study were presented in Table 1. It was determined that all variables had a normal distribution because their kurtosis and skewness values fell within acceptable ranges (53). Before the analysis, the assumptions related to multivariate statistics were checked. It was observed that all Mahalanobis distances were below 15. Multicollinearity was assessed through the Variance Inflation Factor (VIF) and tolerance values. Since the VIF values were below 10 and the tolerance values were above 0.10, this indicated no issues with multicollinearity or redundancy. Consequently, all assumptions were met according to the recommendations of Field (54). Therefore, the Maximum Likelihood (ML) estimation method was employed.

**Table 1:** The square root of the average variance extracted (AVE), correlations matrix, CR, and AVE.

Descriptive statistics and reliabilities						Correlations						
	Mean	SD	CR	AVE	√AVE	1	2	3	4	5	6	7
Scales												
1. Internet gaming disorder	3.33	0.83	0.88	0.52	0.72	–						
2. Social anxiety	1.11	0.67	0.95	0.46	0.67	0.51^**^	–					
3. Insomnia severity	2.91	0.98	0.88	0.45	0.67	0.41^**^	0.62^**^	–				
4. Weekly physical activity (days)	2.15	1.56	–	–	–	−0.10^*^	−0.28^**^	−0.29^**^	–			
Control variable												
5. Daily gaming duration (hours)	5.93	1.88	–	–	–	0.40^**^	0.36^**^	0.34^**^	−0.13^*^	–		
6. Caffeine consumption frequency	3.43	1.09	–	–	–	0.18^**^	0.14^**^	0.14^**^	0.01	0.66	–	
7. Age	19.01	2.86	–	–	–	−0.22^**^	−0.25^**^	−0.15^**^	0.18^**^	−0.15^*^	0.10^*^	–
Skewness	–	–	–	–	–	−0.95	0.62	−0.21	0.11	0.24	−0.53	0.38
Kurtosis	–	–	–	–	–	0.39	−0.42	−1.23	−0.63	−0.40	−0.36	−1.11

**p < 0.01, *p < 0.05, M, mean; Sd, standard deviation; CR, Composite reliability; AVE, Average variance extracted.

Additionally, Confirmatory Factor Analysis (CFA) was conducted to evaluate the measurement model of the scales. The analysis results indicated that acceptable goodness-of-fit values were achieved [*χ*^2^ (884, *N* = 403) = 1494.952; *p* < 0.01; *χ*^2^/df = 1.69; CFI = 0.92; RMSEA = 0.041 (95% CI: 0.038, 0.045); SRMR = 0.046]. Moreover, composite reliability (CR) values were found to be >0.60, and average variance extracted (AVE) values were > 0.45 (see Table 1). When AVE values are <0.50, CR values can still provide evidence of convergent validity when they exceed 0.60 (55, 56). Furthermore, no excessive multicollinearity or singularity issues were detected, as the correlation values between the variables were below 0.85 (48). Therefore, it can be concluded that convergent validity is established. For discriminant validity, the square roots of AVE values were compared with the correlations among the respective variables. As can be seen in Table 1, the √AVE values are higher than all other correlations among the factors, indicating that discriminant validity is achieved (55).

According to the correlation analysis results, young e-sports players with a high level of internet gaming disorder are observed to have a higher likelihood of experiencing insomnia, greater social anxiety, longer daily gaming (e-sports) duration, and higher coffee consumption. In contrast, their participation in physical activity tends to be lower, and they are generally younger. Additionally, e-sports players who experience more social anxiety tend to exhibit more insomnia, longer daily e-sports gaming, lower participation in physical activity, and younger age.

### 3.2 Moderator analysis

In this study, it is assumed that weekly physical activity will moderate the relationship between internet gaming disorder and social anxiety as well as insomnia among young e-sports players. A path model was created in which internet gaming disorder, social anxiety, participation in weekly physical activity, and insomnia severity were used as latent variables. The analysis resulted in achieving acceptable goodness-of-fit values [*χ*^2^ (2, *N* = 403) = 3.843; *p* < 0.01; *χ*^2^/df = 1.92; CFI = 0.99; RMSEA = 0.048 (95% CI: 0.000, 0.120); SRMR = 0.012]. As assumed, internet gaming disorder shows a significant relationship with social anxiety and insomnia severity. This finding indicates that young e-sports players with high levels of gaming disorder are more likely to experience greater social anxiety and insomnia severity compared to those with low levels of gaming disorder. Additionally, it can be stated that e-sports players with higher levels of social anxiety are more likely to experience insomnia severity compared to those with lower social anxiety. It has been identified that social anxiety mediates the relationship between internet gaming disorder and insomnia severity. The control variables, daily gaming duration, and age, significantly affect social anxiety, while only daily gaming duration significantly affects insomnia severity (Table 2).

**Table 2:** Findings related to direct effects.

*Variables*				% 95 CI				
			*β*	LL	UL	Std. err	*z-*value	P (>|z|)
Direct effect (Step 1)								
Internet gaming disorder	→	Social anxiety	0.39^**^	0.303	0.487	0.04	8.39	0.00
Internet gaming disorder	→	Insomnia severity	0.10^*^	0.005	0.199	0.05	2.05	0.04
Social anxiety	→	Insomnia severity	0.47^**^	0.382	0.558	0.04	10.46	0.00
Weekly physical activity	→	Social anxiety	−0.20^**^	−0.283	−0.128	0.03	−5.20	0.00
Weekly physical activity	→	Insomnia severity	−0.13^**^	−0.213	−0.062	0.03	−3.58	0.00
Control variables								
Daily gaming duration	→	Insomnia severity	0.10^*^	0.008	0.199	0.04	2.11	0.03
Caffeine consumption frequency	→	Insomnia severity	0.04	−0.037	0.124	0.04	1.06	0.28
Age	→	Insomnia severity	0.01	−0.064	0.099	0.04	0.42	0.67
Daily gaming duration	→	Social anxiety	0.15^**^	0.066	0.246	0.04	3.39	0.00
Age	→	Social anxiety	−0.11^**^	−0.198	−0.032	0.04	−2.72	0.00

**p < 0.01, *p < 0.05, Std. Err, Standard error; Standardized beta coefficients (β) are reported.

**Figure 2:**
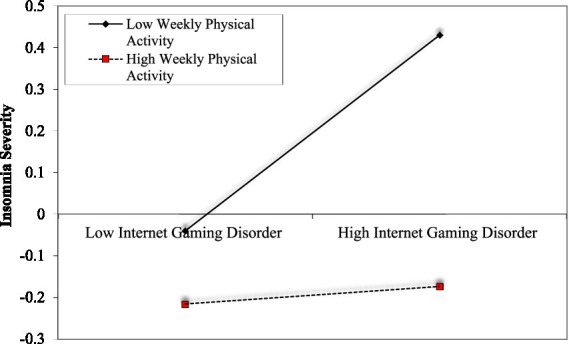
The moderator’s role of weekly physical activity in the relationship between internet gaming disorder and insomnia severity.

**Figure 3:**
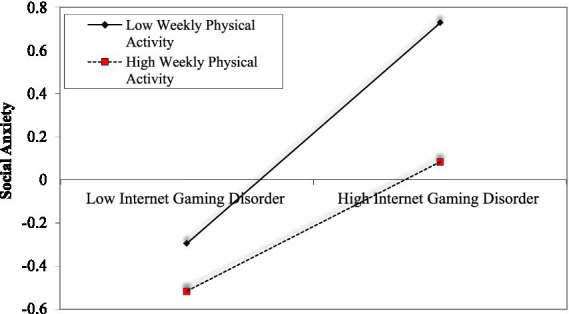
The moderator’s role of weekly physical activity in the relationship between internet gaming disorder and social anxiety.

Table 3 presents all moderator effects. The results indicated that weekly physical activity moderates the relationship between internet gaming disorder and insomnia severity as well as social anxiety. Figure 2 illustrates the simple slopes for high and low weekly physical activity levels. The analysis indicated that e-sports players who participated in less physical activity every week exhibited a significant relationship between high levels of internet gaming disorder and high levels of insomnia severity (*b_simple_* = 0.21, *p* < 0.05, z = 2.91, %95 CI: 0.069, 0.353). In contrast, the relationship between the two variables was insignificant for those engaging in more physical activity (*b_simple_* = −0.00, *p* = 0.90, z = −0.11, %95 CI: −0.133, 0.118). Additionally, it was observed that weekly physical activity negatively predicts insomnia severity. When examining Figure 3, simple slopes for low and high participation in weekly physical activity regarding the relationship between internet gaming disorder and social anxiety are shown. It was predicted that e-sports players with low participation in physical activity would have a significant relationship between high levels of gaming disorder and high levels of social anxiety (*b_simple_* = 0.49, *p* < 0.05, z = 8.06, %95 CI: 0.377, 0.617). For those engaging in higher levels of physical activity, the relationship is also significant, but the effect sizes are lower (*b_simple_* = 0.29, *p* < 0.05, z = 4.79, %95 CI: 0.377, 0.617).

**Table 3:** Findings related to mediator and moderator relationships.

		% 95 CI				
	*β*	LL	UL	Std. err	*z-*value	P (>|z|)
Mediator effect (Step 2)						
Internet gaming disorder → Social anxiety → Insomnia Severity	0.18^**^	0.130	0.241	0.02	6.57	0.00
Moderator effect (Step 3)						
Weekly physical activity × social anxiety → insomnia severity	0.03	−0.065	0.127	0.04	0.63	0.52
Weekly physical activity × internet gaming disorder → insomnia severity	−0.10^*^	−0.201	−0.017	0.04	−2.32	0.02
High weekly physical activity	−0.00	−0.133	0.118	0.06	−0.11	0.90
Low weekly physical activity	0.21^**^	0.069	0.353	0.07	2.91	0.00
Weekly physical activity × internet gaming disorder → social anxiety	−0.10^*^	−0.179	−0.025	0.03	−2.60	0.00
High weekly physical activity	0.29^**^	0.173	0.413	0.06	4.79	0.00
Low weekly physical activity	0.49^**^	0.377	0.617	0.06	8.06	0.00

**p < 0.01, *p < 0.05, Std. Err, Standard Error; Standardized beta coefficients (β) are reported.

## 4 Discussion

Adolescence and young adulthood are critical periods for identity formation (25). During this time, adverse environmental factors, such as IGD, may significantly impact the development of identity (57). Meta-analyses indicate that during these periods, adolescents and young adults frequently experience depression, anxiety, eating disorders (58), and sleep disturbances (59). Additionally, it is known that IGD can lead to certain functional impairments and psychological distress in adolescents and young individuals (60, 61). During this critical developmental period, IGD has the potential to exacerbate mental health issues. As a result, many studies have examined the effects of IGD on adolescents and young adults (58, 59). However, the role of physical activity in influencing certain psychological disorders that IGD may affect during adolescence remains less clear. This may be more pronounced in individuals who engage in prolonged and intensive gaming, such as e-sports players. This study contributes to the literature by examining the relationship between IGD, IS, and SA, and investigating the moderating effects of PA as a potential factor that could reduce the strength of this relationship. The findings indicate that, even after controlling for related variables such as caffeine consumption, age, and daily gaming duration, young individuals exhibiting high levels of IGD are more likely to suffer from higher levels of SA and IS. Young individuals with high levels of IGD and low weekly PA participation are more likely to face higher levels of SA and IS compared to those with high levels of weekly PA participation. Low PA participation predicts a significant increase in both SA and IS as IGD levels rise. However, when weekly PA participation is high, the effect of IGD increase on SA weakens, while its effect on IS diminishes to the point of becoming nonsignificant.

Firstly, the relationship between IGD and IS in young e-sports players was examined. As expected, even after considering control variables (age, caffeine consumption, daily gaming duration), IGD was found to positively and significantly predict IS. The significant impact of IGD on IS is also supported by studies conducted with both e-sports samples and different groups and cultural contexts (13, 37, 59). Melatonin, which is produced in higher amounts during childhood, begins to be secreted in smaller quantities during adolescence and beyond; this can lead to disruptions in sleep patterns (62). The influence of IGD on adolescents and young adults, who naturally experience changes in their sleep tendencies, may exacerbate sleep disturbances. Furthermore, sleep disorders are frequently associated with disrupted emotional processes (63). According to emotion regulation theory, individuals use various strategies to cope with negative emotional states and attempt to reduce emotional intensity (64). In particular, young people and adolescents who are in the process of developing emotion regulation skills (65) may turn to IGD as a coping strategy to alleviate insomnia resulting from negative emotional states. In this context, IGD can be seen not only as a factor leading to insomnia but also as a coping mechanism for dealing with insomnia and negative emotional states.

In the next phase, the mediating role of SA in the relationship between IGD and IS was examined. First, the relationship between IGD and SA was examined. Even after considering control variables such as daily gaming duration and age, which could affect SA, IGD was found to positively and significantly predict SA. These findings support longitudinal and cross-sectional studies conducted in different sample groups and cultural contexts (66–68). For instance, in the study by Ohayon and Roberts (68), it was found that children with IGD were 3.4 times more likely to experience SA compared to their peers. Furthermore, it is plausible that young individuals, still undergoing social cognitive development, may perceive online gaming as a space free from social pressures, thereby increasing the likelihood of IGD development (69, 70). These findings not only suggest that IGD contributes to an increased risk of SA, but also imply that adolescents with elevated levels of SA may be more inclined to develop IGD, viewing online gaming as an escape mechanism. In this context, the existence of a bidirectional interaction between IGD and SA can be proposed. The findings of the study also reveal that SA positively and significantly predicts IS. Numerous studies in the literature support these findings (14, 22). SA can trigger physiological responses by inducing states of intense stress and anxiety (21). In particular, the desire for social acceptance and deficiencies in individual relationships among young individuals increase social anxiety, which in turn leads to elevated levels of stress hormones such as cortisol (71, 72). This condition can make it difficult to fall asleep and maintain sleep. It is especially likely that young individuals continuously question their social interactions, leading to intense anxiety. Such ruminative thoughts (73) may keep the mind active before sleep, making it more difficult to transition to rest. Consequently, both the findings of this study and previous research suggest that IGD not only directly affects IS but also interacts indirectly through SA.

In the next step, the moderating effect of PA on the relationship between IGD and SA was examined. Research conducted across various sample groups and cultural contexts has shown that PA has a strong buffering effect on SA and significantly reduces its levels (14, 74, 75). In our study, a significant relationship was found between high levels of IGD and SA in young e-sports players with low weekly PA participation. However, in those with high weekly PA participation, the impact of IGD on SA was observed to be reduced by half. This finding is consistent with the “stress-buffering hypothesis,” which explains the reducing effect of physical activity on social anxiety through social support mechanisms (76). Additionally, the reduction of stress hormones such as cortisol and the increase in the release of mood-enhancing neurotransmitters like endorphins and dopamine in participants may serve as a biochemical explanation for this effect (77). These biochemical changes can alleviate the tension caused by SA and may also contribute to the development of self-confidence, self-esteem, and self-worth perceptions, particularly in young individuals (24, 78). The anxiety-reducing effect of PA may help young individuals feel more secure in social interactions, thereby enabling them to manage social anxiety more effectively.

The study also examined whether PA has a moderating effect on the relationships between IGD and IS, and SA and IS. In young e-sports players with low weekly PA participation, a significant relationship was found between high levels of IGD and IS. However, in young e-sports players with high weekly PA participation, the impact of IGD on IS was found to be nonsignificant. Previous studies conducted with different sample groups and age cohorts have yielded results indicating that an increase in PA regulates sleep patterns (79–82). The reason for this may lie in the regulatory effect of PA on sleep hormones, which could enhance sleep quality and expedite the process of falling asleep. Daytime exercise has been shown to increase melatonin secretion, facilitating the transition into sleep at night, while also balancing cortisol levels, thereby reducing the effects of stress, anxiety, and depression (83, 84). Additionally, the increased levels of endorphins and serotonin due to exercise can improve mood and support melatonin production, positively affecting sleep quality and potentially mitigating the negative effects of IGD (85). Through these hormonal effects, regular physical activity can promote a healthier sleep pattern in e-sports players. On the other hand, social cognitive theory posits that human behaviors can be shaped through environmental observations (19). Specifically, young e-sports players may sustain PA through the role models in their environment. As their sleep patterns improve and various stressors decrease, these individuals may continue the cycle of physical activity by increasing their sense of self-efficacy.

Finally, physical activity is generally expected to reduce stress and anxiety levels, thereby improving sleep quality (22, 77). However, in this study, the effect of PA did not significantly mitigate insomnia by reducing the symptoms of SA. A possible explanation for this could be that the levels of PA among the participants were not sufficient to buffer the effects of SA and alter its impact on IS. On the other hand, the presence of other moderating factors, such as psychological and environmental variables, may also be considered. Additionally, individuals with high levels of SA may be more sensitive to external stimuli, which could limit the positive effects of PA. In other words, despite the potential benefits of PA, the constant rumination and stress induced by SA (21, 73) may have made it more difficult for young e-sports players to fall asleep, thereby neutralizing the restorative effects of PA.

This study contributes to understanding the extent to which IGD predicts SA and IS levels in young e-sports players, whether SA acts as a mediator in this relationship, and whether PA levels are effective in reducing SA and IS. However, several limitations should be considered when interpreting these findings. First, this study was conducted with individuals aged 14–24 who have played e-sports for at least 3 h per day over the last 6 months, which means that the findings may differ in individuals with varying levels of gaming activity or in different age and gender groups. Additionally, the cross-sectional design of the study makes it difficult to establish causal relationships and introduces limitations in terms of reliability. Future research may adopt experimental designs to explore the changes and interactions of these relationships over time. An experimental approach could more clearly determine the potential moderating effects of PA on the relationship between IGD, SA, and IS. Future studies could explore the impact of personality traits on IGD levels. Additionally, the role of psychological factors, such as social support and emotional regulation skills, in the severity of social anxiety and insomnia could be investigated in more detail. Furthermore, it may be beneficial to compare the effects of different types of e-sports, such as team sports versus individual sports, on social anxiety and insomnia severity. In our study, all types of e-sports games were comprehensively examined. However, to further investigate the effects of game types, more specific studies focusing on different genres, such as strategy games, action games, and sports simulation games, could be conducted. Such an analysis may allow for more detailed insights and diverse findings. Moreover, additional variables, such as socioeconomic status, family support, and emotional regulation, could further enhance the quality of the study, as discussed in Marelić and Vukušić (86). The relevance of physical activity in improving the health of e-sports players, especially through sociological and subcultural aspects, also remains an important area for further research, though it was not fully addressed in this study and should be considered as a limitation.

This study provides practical implications for designing effective interventions to reduce SA levels and regulate IS in young e-sports players. First, strategies can be developed to mitigate the negative effects of IGD on young e-sports players with IGD. In particular, increasing PA durations and directing adolescent individuals toward team sports could have positive effects on both SA and IS. In this context, e-sports clubs and organizations can develop programs that encourage players to adopt more PA habits, thereby promoting such behaviors. Second, support can be provided to e-sports players with social anxiety by focusing on the development of their social skills and enhancing their ability to cope with stress. This could help increase players’ psychological resilience, assisting them in managing negative emotional states. Additionally, limiting players’ gaming time, guided by family support, can establish healthier gaming habits. This strategy will not only provide a more productive performance environment for e-sports players within the club but also improve players’ psychological health, offering benefits in relation to negative moods, disrupted social relationships, and sleep problems. Such interventions could also contribute to the acquisition of efficient players for the clubs.

## 5 Conclusion

This study aims to enhance the understanding of the relationship between IGD and IS. The findings indicate that IGD positively affects the relationship between SA and IS among young e-sports players, while PA negatively influences this relationship. Additionally, SA mediates the relationship between IGD and IS. PA acts as a moderator in the relationships between IGD and SA, as well as IGD and IS. The results demonstrate that PA reduces SA and nearly nullifies the negative effects of IGD on IS. This findings point highlights the beneficial impact of physical activity on the psychological health of young e-sports players. In this regard, future interventions should focus on strategies that psychologically empower young e-sports players and include programs involving both social and physical activities. Families and clubs can adopt such approaches to enhance players’ psychological wellbeing, fostering healthier individuals while also improving players’ productivity.

## Data Availability

The raw data supporting the conclusions of this article will be made available by the authors, without undue reservation.
